# *Plasmodium falciparum *proteome changes in response to doxycycline treatment

**DOI:** 10.1186/1475-2875-9-141

**Published:** 2010-05-25

**Authors:** Sébastien Briolant, Lionel Almeras, Maya Belghazi, Elodie Boucomont-Chapeaublanc, Nathalie Wurtz, Albin Fontaine, Samuel Granjeaud, Thierry Fusaï, Christophe Rogier, Bruno Pradines

**Affiliations:** 1 Unité de Recherche en Biologie et Epidémiologie Parasitaires, Institut de Recherche Biomédicale des Armées, Antenne de Marseille, BP 60109, 13262 Marseille Cedex 07, France; 2 Unité de Recherche en Maladies Infectieuses et Tropicales Emergentes, UMR 6236, Marseille, France; 3 Centre d'Analyses Protéomiques de Marseille, IFR 137, Marseille, France; 4 Unité de Recherche en Physiologie et Pharmacologie Parasitaires, Institut de Recherche Biomédicale des Armées, Antenne de Marseille, Institut de Médecine Tropicale du Service de Santé des Armées, Marseille, France; 5 Technological Advance for Genomics and Clinics, INSERM U928, parc scientifique de Luminy, Marseille, France

## Abstract

**Background:**

The emergence of *Plasmodium falciparum *resistance to most anti-malarial compounds has highlighted the urgency to develop new drugs and to clarify the mechanisms of anti-malarial drugs currently used. Among them, doxycycline is used alone for malaria chemoprophylaxis or in combination with quinine or artemisinin derivatives for malaria treatment. The molecular mechanisms of doxycycline action in *P. falciparum *have not yet been clearly defined, particularly at the protein level.

**Methods:**

A proteomic approach was used to analyse protein expression changes in the schizont stage of the malarial parasite *P. falciparum *following doxycycline treatment. A comparison of protein expression between treated and untreated protein samples was performed using two complementary proteomic approaches: two-dimensional fluorescence difference gel electrophoresis (2D-DIGE) and isobaric tagging reagents for relative and absolute quantification (iTRAQ).

**Results:**

After doxycycline treatment, 32 and 40 *P. falciparum *proteins were found to have significantly deregulated expression levels by 2D-DIGE and iTRAQ methods, respectively. Although some of these proteins have been already described as being deregulated by other drug treatments, numerous changes in protein levels seem to be specific to doxycycline treatment, which could perturb apicoplast metabolism. Quantitative reverse transcription polymerase chain reaction (RT-PCR) was performed to confirm this hypothesis.

**Conclusions:**

In this study, a specific response to doxycycline treatment was distinguished and seems to involve mitochondrion and apicoplast organelles. These data provide a starting point for the elucidation of drug targets and the discovery of mechanisms of resistance to anti-malarial compounds.

## Background

The parasitic protozoon *Plasmodium falciparum *is responsible for approximately 247 million cases of malaria and one million deaths each year, particularly in sub-Saharan Africa [1]. Anti-mosquito measures and new artemisinin-containing treatments have been recently adopted in hopes of achieving the global eradication of malaria. Novel drugs, vaccines and insecticides, as well as deeper insights into parasite biology, human immunity, and vector behaviour, are essential to support these efforts [2].

Over the past 30 years, experimental observations obtained *in vitro *and in clinical studies have demonstrated the anti-malarial activity of tetracycline and its derivatives [3]. Daily doxycycline (DOX) has been shown to be an effective chemoprophylactic in Thailand [4], Indonesia [5], and Kenya [6]. DOX is currently one of the recommended chemoprophylactic regimens for travellers visiting malaria endemic areas in Southeast Asia, Africa and South America [7]. DOX is now recommended by the French Consensus Conference for chemoprophylaxis in countries with a high prevalence of *P. falciparum *resistance to chloroquine or multiple drugs [8]. However, while no instances of *P. falciparum *malaria clinical failure with DOX have been reported yet, three different phenotypes (low, medium and high DOX susceptibility groups) have been identified among *P. falciparum *clinical isolates [9]. These different phenotypes have been associated with *pfmdt *and *pftetQ *copy number variations and *pftetQ *sequence polymorphisms [10].

DOX has long been known to inhibit protein synthesis in bacteria [11] by binding the S4, S7, S9 and S17 proteins of the small 30S ribosomal subunit and various ribonucleic acids of the 16S rRNA, which prevents the binding of aminoacyl transfer RNA to site A of the ribosome [12]. In *P. falciparum*, tetracyclines have been reported to directly inhibit mitochondrial protein synthesis [13] and also to decrease dihydroorotate dehydrogenase activity, which is involved in *de novo *pyrimidine synthesis [14]. DOX inhibits *P. falciparum *synthesis of nucleotides and deoxynucleotides [15]. Minocycline, another tetracycline derivative, also decreases the transcription of mitochondrial genes and plastid genes, indicating that it may target these two organelles [16]. More recently, two research groups [17,18] reported specific action by cyclines on the apicoplast of *P. falciparum *via cell biology and transcriptome approaches. Collectively, these published data indicate that organelles from *P. falciparum *seem to be primary targets for cyclines; however, the molecular mechanisms involved in this plastid regulation are not yet clearly defined, particularly at the protein level.

Proteome studies have contributed substantially to our understanding of parasite biology and host-parasite interactions [19]. Mass spectrometry (MS) methods have been used to enable large-scale identification of proteins at different stages of the malarial parasite life cycle [20,21]. However, few proteomic analyses have been undertaken to better understand the mechanisms of drug action or resistance in *P. falciparum*. The effects of chloroquine and artemisinin derivatives on *P. falciparum *have been studied using different proteomic techniques, such as a gel-based approach [22], SELDI (Surface Enhanced Laser Desorption Ionization) TOF (Time of Flight) MS analysis [23] and, more recently, isoleucine-based SIL (Stable Isotope Labelling) [24]. Until now, *P. falciparum *proteome response following doxycycline treatment has not been studied.

The present study aimed to highlight the metabolic pathways that are affected in *P. falciparum *following DOX treatment. To accomplish this objective, two complementary proteomics approaches were used: two-dimensional fluorescence difference gel electrophoresis (2D-DIGE) and isobaric tagging reagents for relative and absolute quantification (iTRAQ). The combination of these two technologies allowed us to identify proteins that are deregulated in response to doxycycline and were involved in various cellular functions such as redox homeostasis, stress response, protein synthesis, lipid synthesis and energy metabolism. These results indicated that *P. falciparum *organelles seem perturbed by DOX treatment, suggesting that these are the drug's primary targets.

## Methods

### *Plasmodium falciparum *growth conditions and protein extraction

Parasites (chloroquine-resistant W2 clone) were maintained in continuous culture as described elsewhere [25], at 10% haematocrit of type A^+ ^human RBCs suspended in supplemented RPMI 1640 (Invitrogen) and 10% heat-inactivated type A^+ ^human serum at 37°C in a gas mixture of 5% CO_2, _10% O_2 _and 85% N_2_. The medium was changed twice daily. Parasitaemia was monitored daily via microscope by examination of blood smears stained with a RAL^® ^555 kit (Réactifs RAL). Parasite synchronization was performed by sorbitol treatment (D-sorbitol, ICN Biomedicals) as described elsewhere [26]. At the ring stage, parasites were or were not exposed to DOX (Sigma) at 10 μM (the IC_50 _as previously determined [27]) for a period of 24 h. Parasites at the schizont stages during the second cycle after DOX exposure were extracted from the RBCs. Control and treated groups consisted of four biological replicates for the DIGE experiment and three biological replicates for the iTRAQ experiment. IRBCs were washed 3 times in PBS (Invitrogen) and lysed by 0.1% saponin (Sigma) for 5 min. Free parasites were sedimented by centrifugation (9,300 g for 5 min) and washed with PBS 3 times and stored at -80°C. Parasites were resuspended in 10 mM Tris-HCl buffer (pH 8) and disrupted by ultrasonication (Vibracell 72412, Bioblock Scientific) for 5 min on ice at maximum amplitude. After ultracentrifugation (100,000 g for 1 h at 4°C), soluble protein fractions were recovered from the supernatant and the pellet containing membrane protein fractions was then suspended in 4% (w/v) 3-[(3-cholamidopropyl)dimethylamonio]-1-propanesulfonate (CHAPS) (Sigma). All of the fractions were precipitated in 100% acetone (Sigma) to remove lipids, and the protein concentration of each sample was estimated using the Lowry-based DC assay (Biorad) according to the manufacturer's instructions. All of the samples were suspended in standard cell lysis buffer (7 M urea, 2 M thiourea, 4% CHAPS, 30 mM Tris base, pH 8.5 (Sigma)) to obtain a protein concentration adjusted to 2.5 μg/μL.

### 2D-DIGE

Protein samples were minimally labelled with CyDye according to the manufacturer's recommended protocols (GE Healthcare). Briefly, soluble protein samples from the control parasites (50 μg) and the DOX-treated parasites (50 μg) were labelled with 400 pmol of either Cy3 or Cy5 (in four biological quadruplicates, with a dye swap) and an internal standard (50 μg) was labelled with 400 pmol of Cy2, freshly dissolved in N, N-dimethylformamide (DMF) (Sigma), and incubated on ice for 30 min in the dark. The reaction was quenched with 1 μL of free lysine (10 nM) by incubation for 10 min on ice. Cy3-, Cy5- and Cy2-labeled samples were then pooled, and an equal volume of 2 × sample buffer was added (8 M urea, 2 M thiourea, 4% (w/v) CHAPS, 10 mM dithiothreitol (DTT), and 1% (v/v) immobilized pH gradient (IPG) Buffer 3-10 (GE Healthcare). The membrane protein samples were treated as described above with either IPG Buffer 4-7 or IPG Buffer 6-11 (GE Healthcare). The mixture of labelled proteins was then separated by two-dimensional gel electrophoresis (2-DE) (See additional file 1 for more details).

### 2-D Image analysis

Gel images were acquired with a Typhoon^® ^Trio Image scanner (GE Healthcare) at different excitation wavelengths (Cy3, 580 BP 30/green (532 nm); Cy5, 670 BP 30/red (633 nm); Cy2, 520 BP 40/blue (488 nm)). Images were cropped with ImageQuant^® ^software (GE Healthcare) and further analysed using DeCyder v6.5 (GE Healthcare). The software was used to perform gel alignment, spot averaging and normalization and Student's *t*-test to determine which protein spots changed in abundance in response to DOX-treatment. The number of detected spots showing a difference with a *p*-value of < 0.05 was then determined.

### In-gel trypsin digestion

After imaging, the gels were stained either with Sypro Ruby (Bio-Rad) according to the manufacturer's protocol and then scanned using the typhoon scanner or with Coomassie Brilliant Blue (CBB) G-250 as previously described [28]. Spots of interest were manually excised. Protein spots were digested overnight at 37°C with sequencing-grade trypsin (12.5 μg/mL; Promega Madison) in 50 mM NH_4_HCO_3 _(Sigma). The resulting peptides were extracted with 25 mM NH_4_HCO_3 _for 15 min, dehydrated with acetonitrile (ACN) (Sigma), incubated with 5% formic acid (Sigma) for 15 min under agitation, dehydrated with ACN, and finally completely dried using a SpeedVac. Samples were then stored at -20°C before analysis by MS.

### iTRAQ labelling and strong cation exchange

After protein precipitation in acetone, the samples were dissolved in 20 μL of dissolution buffer, reduced, alkylated, trypsin-digested and labelled using the iTRAQ reagents four-plex kit according to the manufacturer's instructions (Applied Biosystems). The resulting peptide solutions from control and DOX-treated soluble protein samples were labelled with iTRAQ114 and iTRAQ117, respectively, and incubated at room temperature for 1 h. Labelled peptides were then pooled and acidified by mixing with the cation buffer load iTRAQ reagent for a total volume of 1 ml. The peptide mixture was subsequently fractionated by strong cation exchange (SCX) chromatography (See additional file 1 for more details). The elution was monitored by absorbance at 214 and 280 nm (Additional file 2), and 40 fractions were collected. These experiments were conducted in three different biological replicates. The same protocol was applied to the membrane proteins samples, but labelling was done with iTRAQ115 (control) and iTRAQ116 (DOX-treatment). Each fraction of iTRAQ-labelled sample was dried using a Speedvac, reconstituted in 12 μL of buffer (1% v/v formic acid in H_2_O) and analysed by nano-liquid chromatography tandem mass spectrometry (nano-LC-MS/MS).

### Protein identification by nano-LC MS/MS

Protein digests extracted from excised DIGE gel spots were analysed by nano-LC-ESI-MS/MS. Purification and analysis were performed on a C18 capillary column using a CapLC system (Waters) coupled to a hybrid quadrupole orthogonal acceleration time-of-flight tandem mass spectrometer (Q-TOF Ultima, Waters). Chromatographic separations were conducted on an RP capillary column (AtlantisTM dC18, 3 μm, 75 μm × 150 mm Nano EaseTM, Waters) with a 180-200 nl.min^-1 ^rate of flow (See additional file 1 for more details).

### DIGE protein database search

The data were searched using Mascot software against the *P. falciparum *National Center for Biotechnology Information non-redundant protein database (NCBInr, NIH, Bethesda, MD, March 27^th^, 2008). Search parameters allowed for one missed tryptic cleavage site, the carbamidomethylation of cysteine, and the possible oxidation of methionine; the precursor and product ion mass error tolerance was < 0.2 Da. All identified peptides had a Mascot score greater than 28 (*P. falciparum*, 12,220 sequences), corresponding to a statistically significant (*p *< 0.05) confident identification. Moreover, among the positive matches, only protein identifications based on at least two different non-overlapping peptide sequences of more than six amino acids and with a mass tolerance < 0.05 Da were accepted (Additional file 3). These additional validation criteria struck a balance that limited the number of false positive matches without missing real proteins of interest.

### iTRAQ protein database search and quantification

Mascot distiller software (v2.1.1, Matrix Science) was used to convert MassLynx.raw MS/MS data files into mascot generic files (mgf). (See additional file 1 for more details). For protein identification, mgf data files were searched against a mixed file of *Homo sapiens *and *P. falciparum *sequences in the NCBInr (NIH, Bethesda, MD) protein database (229,804 sequences on September 12^th^, 2008 for soluble proteins and 230,260 sequences on October 21^st^, 2008 for membrane proteins) using the MASCOT algorithm (v2.2, Matrix Science). (See additional file 1 for more details). For protein quantification, data analysis was performed with Multi-Q 1.6.1.1. as described elsewhere [29]. The MassLynx.raw data files from the Q-TOF Ultima (Waters) were previously converted into files of the mzXML format by the massWolf program [30]. (See additional file 1 for more details). Geometric means of the ratios of DOX-treated protein to control protein (iTRAQ117/iTRAQ114 for soluble proteins and iTRAQ116/iTRAQ115 for membrane proteins) and the standard deviation were calculated. Proteins with ratios ≤ 0.80 or ≥ 1.20 between DOX-treated and untreated experimental conditions were considered regulated proteins, as reported elsewhere [31].

### Bioinformatics predictions of biological processes and subcellular localization of identified proteins

The NCBI GI (GeneInfo Identifier) numbers of *P. falciparum *proteins identified were converted into standard gene names for retrieval from the UniprotKB ID module [32]. Then, in UniprotKB, PlasmoDB accession numbers of proteins were retrieved for further analysis. Biological processes and subcellular localization of differentially expressed proteins were assessed using Gene Ontology annotations downloaded from PlasmoDB [33]. The transit peptides that enable proteins to target the apicoplast were identified by the PlasmoAP tool [34].

### Quantitative real-time RT-PCR

The same parasite cultures used for the proteomic analysis were also used to perform quantitative RT-PCR. Total RNA was extracted with TRIZOL^® ^reagent following the manufacturer's recommendations (Invitrogen) and treated with DNase (DNAfree^®^, Ambion). Total RNA was quantified with the NanoDrop ND-1000 (Labtech), followed by quality assessment with the 2100 Bioanalyzer (Agilent Technologies) according to the manufacturer's protocol. Acceptable A260/A280 ratios were in the range of 1.8-2.2. Acceptable rRNA ratios (28S/18S) needed to be > 0.9, and RIN (RNA Integrity Number) values needed to be > 8.0. Total RNA (1 μg) was reverse transcribed with the High-Capacity cDNA Archive Kit as described by the manufacturer (Applied Biosystems). The primer pairs used (Eurogentec), (Additional file 4) were designed with Primer Express software v2.0 (Applied Biosystems). Real-time transcript quantification was performed using a 7900HT Fast Real-Time PCR system (Applied Biosystems). Amplification reactions and the 2^-ΔΔCt ^method of relative quantification to estimate relative expression of mRNA targets were performed as previously described [35]. All data were expressed as means ± standard deviation. A two-tailed Student's *t*-test was employed to compare RT-PCR gene expression levels. Statistical significance was defined as *p *< 0.05.

## Results

### Phenotypic effect of DOX treatment

Ring stage parasites (> 95%) were incubated with 10 μM DOX (*i.e*. corresponding to IC_50_) for 24 h followed by a chase period until the end of the successive cycle at 84 h. Then, parasites were collected at the schizont stage for several reasons. First, DOX exerts delayed effects against *P. falciparum *[36], *i.e*. exposing the parasites to DOX does not lead to phenotypic effects at the end of the first cycle but instead at the end of the successive cycle. A chase period was applied because continuous DOX exposure leads to almost 100% parasite lethality. Secondly, this drug has an increased potency against the schizont stage [17], and finally, protein synthesis in parasites is maximal during trophozoïte and schizont stages [37]. At the beginning of the experiments, all cultures had a parasitaemia equal to 2%. At the end of the experiments, parasitaemia obtained from untreated and DOX-treated iRBCs were 7.9% ± 0.7 and 3.7% ± 0.5, respectively. Both control and treated cultures were at the schizont stage (> 90%).

### *Plasmodium falciparum *response to DOX treatment according to 2D-DIGE analysis

**Figure 1:**
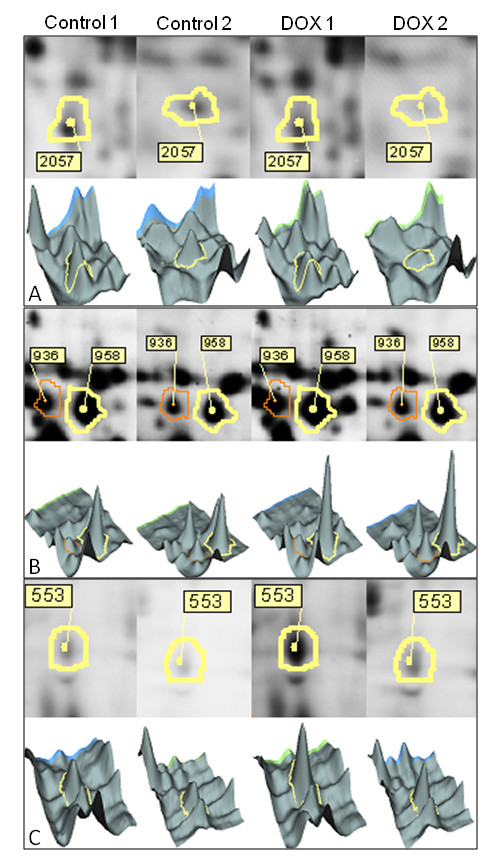
**Examples of 2D and 3D representations of fluorescence intensity of spots that were up and down regulated in response to DOX treatment, as visualized with Decyder software in 2D-DIGE analysis**. (A) In the soluble proteins gel with pI 3-10, spot 2057 was down regulated under DOX treatment and was identified as a putative protein: proteasome subunit alpha type 5 (PF08_0109). (B) In the membrane proteomic map with pI 4-7, spots 936 and 958 were up regulated under DOX treatment and identified as isoforms of the same putative protein: TCP-1/cpn60 chaperon family (MAL13P1.283). (C) In the membrane proteomic map with pI 6-11, spot 553 was up regulated and identified as a putative pyruvate kinase (PFF1300w).

Since membrane-associated proteins are generally under-represented by two-dimensional electrophoresis, proteins extracted from the schizont stages were separated into soluble and membrane fractions to circumvent this limitation. Then, four biological replicates from untreated or DOX-treated samples were divided into soluble or membrane protein fractions and their protein expression profiles were compared using 2D-DIGE methods. After imaging, DeCyder software was used to detect spot levels that were significantly deregulated by DOX treatment (Figure 1 and additional file 5). Among the three type of gels (with 18-cm 3-10, 4-7 and 6-11 linear IPG strips), a total of 150 spots were considered to be significantly deregulated (45, 45 and 60 spots respectively in the membranaire protein gels with 4-7 linear IPG strip, in the membranaire protein gels with 6-11 linear IPG strip and in the soluble protein gels with 3-10 linear IPG strip); 95 spots (63%) were up-regulated and 55 (37%) spots down-regulated in response to DOX treatment (*p *< 0.05, Student's *t*-test, and spot ratios ≤ 0.74 or ≥ 1.35). Thirty five spots (11, 12 and 12 spots respectively) could not be excised manually (invisible after gel coloration compared to scanning images with Typhoon). Then, 115 spots were submitted to identification by nano LC MS/MS (34, 33 and 48 spots respectively). Sixty seven spots were identified by mass spectrometry (MS) (15, 20 and 32 spots respectively). Forty eight spots were not identified (19, 13 and 16 spots respectively) and 20 spots (5, 9 and 6 spots respectively) were not retained, because comigration of proteins confounded the ability to identify individual proteins. Finally, 47 protein spots were identified (Table 1). However, some proteins were detected in more than one spot, indicating different isoforms. A total of 32 distinct proteins, according to their accession numbers, were finally identified (Figure 1) (Table 1, peptide details in Additional file 2). Among these proteins, 22 were up-regulated, and 10 were down-regulated. They were classified according to their biological functions in Table 1. Some proteins were identified as being involved in primary metabolism (*e.g*., carbohydrate, protein and amino-acid metabolism, and DNA replication). Proteins involved in anti-oxidative stress (PF08_0131, 1-Cys peroxiredoxin in spot 2251 and PF14_0368, 2-Cys peroxiredoxin in spot 1821) and two proteins with unknown functions were also identified. Several isoforms of the same protein (PF10_0155, enolase in spots 1066 and 1089; PF14_0598, glyceraldehyde-3-phosphate dehydrogenase in spots 1288, 1295 and 1325 in Figure 1A and Table 1) were identified in adjacent spots.

**Table 1:** Differentially expressed proteins in DOX-treated parasites (DIGE quantification).

Accession number	Name	Spot	MASCOT score	Peptides matched	**Ratios**^**a**^	**Biological process**^**b**^	**Predicted Localization**^**c**^	**Fraction**^**d**^
PF13_0304	Elongation factor 1 alpha	1078	236	5	2.85*	Translation	Cytoplasm	S
		1111	226	4	2.65*			S
		1143	246	5	2.42*			S
		1026	309	6	2.31*			S
		848	411	9	2.04**			M (2)
		833	306	6	2.03**			M (2)
		1168	254	6	1.65*			S
PF13_0143	Phosphoribosylpyrophosphate synthetase	964	174	3	2.36*	Carbohydrate metabolism	Apicoplast	M (2)
PFB0445c	DEAD box helicase, UAP56	2246	138	2	2.25*	Nucleic acid binding	Unknown	S
PFI1105w	Phosphoglycerate kinase	1025	515	10	2.14*	Carbohydrate metabolism	Unknown	S
		993	343	6	2.07*			S
PF14_0598	Glyceraldehyde-3-phosphate dehydrogenase	1288	114	2	2.13*	Carbohydrate metabolism	Cytoplasm	S
		1295	276	5	1.89*			S
		1325	472	8	1.81*			S
**PFI1090w**	**S-adenosylmethionine synthetase**	**1236**	**88**	**2**	1.99*	**One carbon compound metabolism**	**Cytoplasm**	**M (1)**
PFF1300w	Putative pyruvate kinase	800	217	4	1.95*	Carbohydrate metabolism	Apicoplast	S
		788	120	3	1.86*			S
		1672	110	2	1.70*			M (1)
		553	149	3	1.61**			M (2)
PF14_0368	2-Cys peroxiredoxin	1821	116	2	1.94*	Anti oxidative stress	Unknown	M (2)
PFE0690c	Rab1 protein	2221	171	4	1.89**	Intracellular protein transport	Cytoplasm	S
PF11_0396	Protein phosphatase 2C	2309	144	2	1.84*	Protein amino acid dephosphorylation	Cytoplasm	S
**PFF1335c**	**4-methyl-5(B-hydroxyethyl)-thiazol Monophosphate biosynthesis enzyme**	**2198**	**282**	**5**	1.69*	**Thiamin biosynthesis**	**Cytoplasm**	**S**
MAL13P1.283	TCP-1/cpn60 chaperonin family	958	828	13	1.64*	Protein folding	Cytoplasm	M (1)
		936	315	6	1.38*			M (1)
**PFF1155w**	**Hexokinase**	**795**	**123**	**3**	1.61*	**Carbohydrate metabolism**	**Unknown**	**S**
PF14_0425	Fructose-bisphosphate aldolase	1180	552	10	1.60***	Carbohydrate metabolism	Unknown	S
		917	561	10	1.43*			M (2)
PF14_0076	Plasmepsin 1 precursor	1406	215	4	1.58***	Haemoglobin Catabolism	Membrane	S
**MAL8P1.69**	**14-3-3 protein**	**1647**	**71**	**2**	1.54*	**Protein folding**	**Unknown**	**S**
**PF08_0131**	**1-Cys peroxiredoxin**	**2251**	**83**	**2**	1.52*	**Anti oxidative stress**	**Unknown**	**M (1)**
**PF08_0074**	**DNA/RNA-binding Protein Alba**	**1237**	**113**	**3**	1.51*	**Nucleic acid binding**	**Nucleus**	**M (2)**
**PF14_0486**	**Elongation factor 2**	**1264**	**164**	**4**	1.48*	**Translation**	**Cytoplasm**	**S**
PF13_0214	Elongation factor 1-gamma	1984	77	2	1.47*	Translation	Apicoplast	S
PF10_0155	Enolase	1066	439	8	1.43**	Carbohydrate metabolism	Cytoplasm	M (1)
		1089	584	10	1.35*			M (1)
PFL0960w	D-ribulose-5-phosphate 3-epimerase	2248	94	2	1.35**	Carbohydrate metabolism	Unknown	M (1)
PF11_0117	Replication factor C subunit 5	1020	106	2	0.70*	DNA replication	Nucleus	M (2)
PF08_0109	Proteasome subunit alpha type 5	2057	79	2	0.63*	Ubiquitin dependent protein catabolism	Unknown	S
PFL0185c	Nucleosome assembly protein 1	967	170	4	0.61**	Nucleosome assembly	Nucleus	S
PF11_0313	60S ribosomal protein P0	2081	100	2	0.60*	Translation	Mitochondrion	M (1)
PF11_0282	Deoxyuridine 5'-triphosphate nucleotidohydrolase	2332	306	5	0.60*	DNA replication	Nucleus	S
**MAL8P1.95**	**Conserved *Plasmodium *protein**	**1532**	**241**	**3**	0.58*	**Unknown**	**Unknown**	**S**
PF14_0678	Exp-2	1421	153	3	0.57**	Unknown	Membrane	M (1)
PF11_0183	GTP-binding nuclear protein ran/tc4	1451	98	2	0.49*	Nucleus transport	Nucleus	M (2)
PF13_0033	26S proteasome regulatory subunit	854	341	8	0.47*	Ubiquitin dependent protein catabolism	Nucleus	M (2)
PF14_0359	HSP40, Subfamily A	648	204	4	0.35*	Protein folding	Unknown	M (2)

In bold: differentially expressed proteins commonly identified in DIGE and iTRAQ. ^a^Significant modification in protein ratios between DOX and control cells with * *P *< 0.05, ** *P *< 0.01, *** *P *< 0.001, Student's *t*-test. ^b^Data depicted from PlasmoDB (gene ontology biological process annotation). ^c^Data depicted from PlasmoDB (gene ontology location annotation or PlasmoAP to predict apicoplast addressing). ^d^S = soluble, pI 3-10; M = membrane (1) corresponds to pI 4-7 and (2) to pI 6-11.

### *Plasmodium falciparum *response to DOX treatment according to iTRAQ analysis

The same soluble and membrane protein extracts used for 2D-DIGE analysis were subjected to iTRAQ analysis. For this analysis, three biological replicates from untreated or DOX-treated parasites were digested, and the peptides were labelled with different isobaric tags. Of the soluble protein samples, 422 proteins were confidently identified including 246 plasmodial proteins (58.3%) and 176 human proteins (41.7%) in the three biological replicates. Among them, 169 were quantified (*i.e*., with at least 4 labelled, non-degenerated peptides), including 14 human proteins (8.3%) and 155 plasmodial proteins (91.7%). Twenty-two proteins displayed significant differences in expression levels (proteins with fold change ≤ 0.80 or ≥ 1.20 were considered as differentially expressed proteins); 18 of these proteins were up-regulated, and four were down-regulated following DOX exposure (Table 2). In membrane protein samples, 308 proteins were confidently identified, including 204 plasmodial proteins (66.2%) and 104 human proteins (33.8%) in the three biological replicates. Among them, 156 were quantified, including 9 human proteins (5.8%) and 147 plasmodial proteins (94.2%). Eighteen proteins displayed significant differences in expression; 14 of these proteins were up-regulated and four were down-regulated following DOX exposure. In total, 40 proteins were differentially expressed in response to DOX treatment (Table 2); 80% were up-regulated (32 out of 40), and 20% were down-regulated (8 out of 40). The proteins up-regulated by DOX treatment were associated with haemoglobin catabolism, protein synthesis, protein processing, anti-oxidative stress and phospholipid metabolism. Down-regulated proteins were mostly associated with protein synthesis/processing or nuclear transport. A substantial proportion of the proteins (20%, 8 out of 40) have not been assigned to a biological function yet. Two up-regulated proteins were identified as human proteins: biliverdine reductase and S100-calcium binding protein A4. Their function and location in human cells were precised in Table 2.

**Table 2:** Differentially expressed proteins in DOX-treated parasites (iTRAQ quantification).

**Accession number**^**a**^	Name	Peptides quantified	Ratios ± SD	**Biological process**^**b**^	**Predicted Localization**^**c**^
PF13_0130	Vacuolar ATP synthase subunit g	3	2.47 ± 1.07	Vacuolar acidification	Membrane
PFC0735w	40S ribosomal protein S15A putative	3	1.93 ± 0.71	Translation	Cytoplasm
**PF08_0074**	**DNA/RNA-binding Protein Alba, putative**	**6**	**1.82 ± 0.08**	**Nucleic acid binding**	**Nucleus**
MAL13P1.214	Phosphoethanolamine N-methyltransferase	6	1.79 ± 0.47	Phosphatidylcholine biosynthesis	Unknown
PF10_0068	RNA binding protein putative	3	1.76 ± 0.16	Nucleic acid binding	Apicoplast
PFB0340c	Serine repeat antigen 5 (SERA-5)	8	1.76 ± 0.37	Proteolysis	Unknown
PFC0920w	Histone H2A variant putative	3	1.59 ± 0.25	Nucleosome assembly	Apicoplast
**PFI1090w**	**S-adenosylmethionine synthetase**	**5**	**1.57 ± 0.25**	**One carbon compound metabolism**	**Cytoplasm**
PFB0915w	Liver stage antigen-3	4	1.54 ± 0.04	Unknown	Membrane
PFL1545c	Chaperonin cpn60	3	1.52 ± 0.40	Protein folding	Apicoplast
PFF0835w	Conserved *Plasmodium *protein	5	1.50 ± 0.01	Unknown	Unknown
PF08_0110	Rab18 GTPase	3	1.49 ± 0.13	Intracellular protein transport	Cytoplasm
PF14_0078	Plasmepsin III|HAP protein	6	1.48 ± 0.04	Haemoglobin Catabolism	Membrane
PF14_0324	Hsp70/Hsp90 organizing protein putative	17	1.46 ± 0.21	Protein folding	Unknown
PF10_0115	QF122 antigen	9	1.45 ± 0.04	Nucleic acid binding	Apicoplast
PF14_0655	RNA helicase-1 putative	5	1.44 ± 0.01	Translation	Cytoplasm
PF14_0201	Surface protein putative Pf113	6	1.44 ± 0.07	Unknown	Membrane
**PF14_0486**	**Elongation factor 2**	**16**	**1.41 ± 0.30**	**Translation**	**Cytoplasm**
PF10_0323	Early transcribed membrane protein 10.2	4	1.40 ± 0.08	Unknown	Membrane
PFL1170w	Polyadenylate-binding protein putative	12	1.35 ± 0.05	Transcription	Unknown
gi|544759	Biliverdin reductase B	2	1.35 ± 0.16	Porphyrin metabolism	Cytoplasm
PFE0870w	Transcriptional regulator putative	4	1.34 ± 0.10	Transcription	Nucleus
gi|4506765	S100 calcium-binding protein A4	2	1.34 ± 0.06	Cell growth	Cytoplasm
PF11_0062	Histone H2B	7	1.34 ± 0.12	Nucleosome assembly	Apicoplast
PF14_0391	60S ribosomal protein L1 putative	2	1.32 ± 0.13	Translation	Cytoplasm
**MAL8P1.69**	**14-3-3 protein**	**6**	**1.30 ± 0.19**	**Protein folding**	**Unknown**
MAL13P1.56	M1-family aminopeptidase	5	1.28 ± 0.06	Haemoglobin Catabolism	Apicoplast
PF14_0439	Leucine aminopeptidase putative	4	1.27 ± 0.18	Haemoglobin Catabolism	Apicoplast
PF08_0096	RNA helicase putative	3	1.24 ± 0.04	Transcription	Unknown
**PF08_0131**	**1-Cys peroxiredoxin**	**9**	**1.22 ± 0.15**	**Anti oxidative stress**	**Unknown**
PFE0585c	Myo-inositol 1-phosphate synthase putative	10	1.22 ± 0.03	Phospholipid biosynthetic process	Unknown
PFL1720w	Serine hydroxymethyltransferase	3	1.21 ± 0.01	One carbon compound metabolism	Unknown
PF14_0167	Prefoldin subunit 2 putative	4	0.80 ± 0.05	Protein folding	Cytoplasm
PFI1780w	*Plasmodium *exported protein (PHISTc)	2	0.80 ± 0.19	Unknown	Apicoplast
**MAL8P1.95**	**Conserved *Plasmodium *protein**	**5**	**0.76 ± 0.09**	**Unknown**	**Unknown**
PFE0290c	Conserved *Plasmodium *protein	3	0.75 ± 0.06	Unknown	Unknown
PFA0110w	DnaJ protein putative	5	0.74 ± 0.24	Protein folding	Membrane
PF08_0087	Importin alpha putative	5	0.73 ± 0.12	Nucleus transport	Nucleus
PFD0090c	*Plasmodium *exported protein (PHISTa)	4	0.61 ± 0.14	Unknown	Apicoplast
PF11_0351	Heat shock protein hsp70 homologue	6	0.54 ± 0.04	Protein folding	Mitochondrion

In bold: differentially expressed proteins commonly identified in DIGE and iTRAQ. ^a^PlasmoDB accession number, except for two human proteins (NCBI accession number). ^b^Data depicted from PlasmoDB (gene ontology biological process annotation). ^c^Data depicted from PlasmoDB (gene ontology location annotation or PlasmoAP to predict apicoplast addressing).

### Comparison of differentially expressed proteins identified by 2D-DIGE and iTRAQ

**Figure 2:**
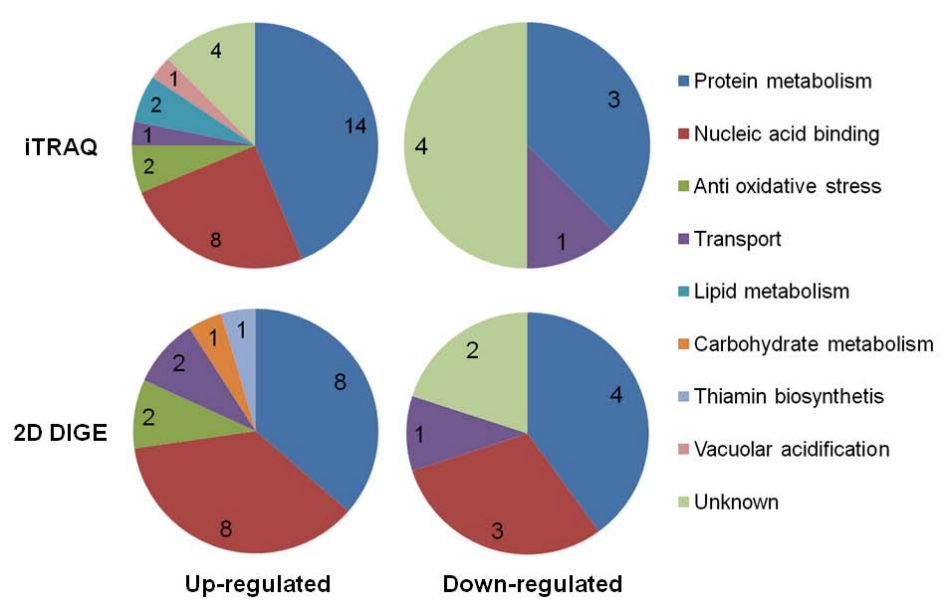
**Functional classification of differentially expressed proteins under DOX treatment identified in iTRAQ and DIGE experiments**.

Of the deregulated proteins identified by DIGE, 19% (six out of 32) were also identified by iTRAQ. The proteins identified by both approaches (DIGE and iTRAQ) were similarly deregulated; for example, S-adenosylmethionine synthetase (DIGE fold change of 1.99 in Table 1 and 1.57 with iTRAQ in Table 2) and 1-Cys peroxiredoxin (fold change of 1.52 with DIGE and 1.22 with iTRAQ). The metabolic processes in which the proteins are involved are similar and, for the most part, changes in protein expression levels were similar between the two approaches (Figure 2). The main metabolic systems identified by both proteomics approaches were protein metabolism, the anti-oxidant response mechanism, nucleic acid binding and transport mechanisms. Both approaches revealed a down-regulation of proteins involved in protein synthesis metabolism and transport mechanism and an up-regulation of proteins involved in protein metabolism and anti-oxidant response mechanisms (Figure 2). However, proteins involved in nucleic acid binding were characterized differently by the two methods; these proteins were up-regulated according to the iTRAQ data and down-regulated according to the DIGE data. Proteins involved in carbohydrate metabolism were all up-regulated and only identified by the DIGE method (Table 1). In addition, proteins involved in specific metabolic pathways were only identified by iTRAQ (Figure 2 and Table 2). Those proteins identified by iTRAQ alone were either membrane proteins involved in vacuolar acidification or enzymes involved in phospholipid metabolism (Table 2). Fifteen soluble proteins were identified in the soluble fractions but not in the membrane fractions (Table 1). Fourteen proteins were identified only in membrane fractions, but only two are actually thought to belong to the membrane, calling into question the effectiveness of the 2D-PAGE approach for characterising membrane proteins. The iTRAQ method, in contrast, allowed identification and quantification of a large number of high molecular weight proteins and membrane proteins (Table 2).

### Subcellular localization of regulated proteins

**Figure 3:**
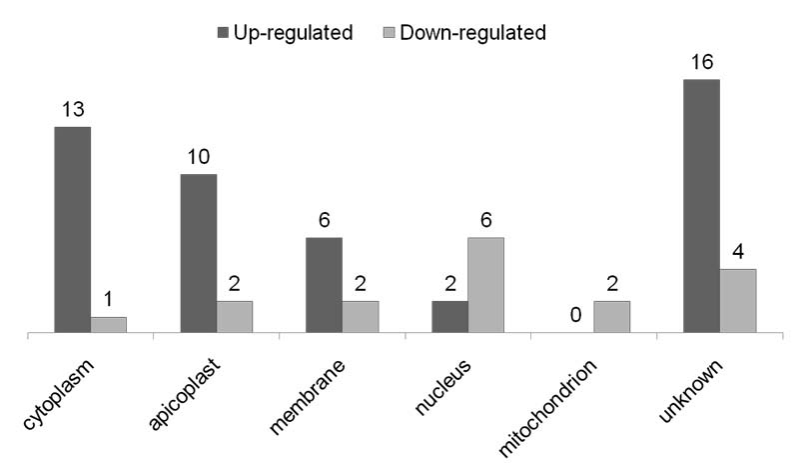
**Number of differentially expressed proteins, identified by iTRAQ and DIGE experiments, corresponding to different functions and subcellular localizations**.

As predicted by gene ontology in PlasmoDB and PlasmoAP (for apicoplast addressing), the cellular localization (Table 1 and Table 2) of the 64 differentially regulated plasmodial proteins identified by the two proteomic approaches was as follows: 21% in the cytoplasm, 19% in the apicoplast, 13% in the membrane, 13% in the nucleus, 5% in the mitochondria and 29% unknown. Cytoplasmic, apicoplastic and membrane proteins were generally up-regulated, while nuclear and mitochondrial proteins were more often down-regulated (Figure 3). All of the identified apicoplastic proteins were encoded by the nuclear genome of *P. falciparum *and not by its plastid genome.

### RT-PCR quantifications

To validate the proteomics results, quantitative RT-PCR was performed because antibodies against *P*. *falciparum *are not commercially available, and the proteomics data suggest that apicoplast function was particularly perturbed by DOX-treatment. Thus, three apicoplast transcripts were chosen to evaluate the modifications observed in this organelle following DOX-treatment. PFI1090w (the S-adenosylmethionine synthetase) and MAL8P1.95 (a conserved *Plasmodium *protein), which had similar expression profiles (up and down regulation respectively) according to both DIGE and iTRAQ, also had similar expression profiles at the transcript level (Table 3). PF14_0439 (leucine aminopeptidase) was shown to be upregulated by iTRAQ analysis and was also up-regulated at the transcript level. Moreover, mRNA for the *PftufA*, *PfsufB *and *PfclpC *proteins encoded by the plastid genome, which were not quantified at the protein level by DIGE or iTRAQ, were down-regulated at the transcript level. Two transcripts of PF07_0033 (CG4) and PFE1195w (Karyopherin beta) proteins were used as positive controls in RT-PCR experiments (Their qRT-PCR ratios were 1.05 ± 0.09 and 0.98 ± 0.12 respectively) because they were not deregulated in iTRAQ proteomic approach (iTRAQ ratios were 1.01 ± 0.03 and 1.07 ± 0.05 respectively), (Table 3).

**Table 3:** Quantitative RT-PCR results.

Accession nr	Description	DIGE ratios	iTRAQ ratios	**qRT-PCR ratios**^**a**^
PFCOMPIRB-TufA	TufA	NA	NA	0.30 ± 0.04
PFCOMPIRB-SufB	SufB	NA	NA	0.32 ± 0.12
PFCOMPIRB-ClpC	ClpC	NA	NA	0.26 ± 0.08
PFI1090w	S-adenosylmethionine synthetase	1.99	1.57 ± 0.25	1.72 ± 0.16
MAL8P1.95	Conserved *Plasmodium *protein	0.57	0.76 ± 0.09	0.44 ± 0.03
PF14_0439	Leucine aminopeptidase putative	NA	1.27 ± 0.18	1.48 ± 0.23
PF07_0033	Cg4 protein	NA	1.01 ± 0.03	1.05 ± 0.09
PFE1195w	Karyopherin beta	NA	1.07 ± 0.05	0.98 ± 0.12

^a^qRT-PCR ratios correspond to the relative expression of target mRNA between the DOX treated and the control (mean of three biological replicates). NA: not available.

## Discussion

In the present study, proteome changes was researched in *Plasmodium falciparum *following DOX treatment in order to clarify the action mechanisms of this drug using two proteomic approaches, 2D-DIGE and iTRAQ. These techniques have been shown to be complementary in studying protein changes as they have distinct physicochemical properties that favour identification of different proteins [38,39]; that is the reason why a 19% overlap was observed between deregulated proteins identified by the both methods. Analysis of the DIGE results detected differentially expressed proteins with PTMs (post translational modifications) after DOX treatment (Table 1 and Figure 1). In particular, enolase and aldolase have previously been reported to possess several differentially expressed isoforms during the schizont stages of *P. falciparum *[40]; these two proteins are localized in Maurer's cleft [41] and in the food vacuole [42]. However, little is known about the role of these PTMs in the plasmodial regulation of protein expression under either physiological conditions or anti-malarial treatment.

Few proteomic studies have been undertaken to elucidate the mechanisms of drug action in *P. falciparum *but all share some common features with the present work [22,24,43]. After exposure to anti-malarial treatment, proteome analysis has generally revealed a low number of differentially expressed proteins with an upregulation of proteins involved in glycolysis, chaperoning or redox metabolism. In a stable isotope labelling experiment after artemisinin and chloroquine treatment in schizont stages of *P. falciparum*, among more than 800 quantified proteins, only 41 and 38 were up-regulated, respectively [24]. However, none of these proteins were associated with heat shock response or glycolysis functions, probably because the design of the study did not allow it, *e.g*. the SILAC method, which explores only newly synthesized proteins, was used. In a gel-based study, arthemeter and lumefantrine treatment were respectively associated with an up-regulation of 22 and 41 proteins [43]. In another study, chloroquine treatment increased the number of oxidized proteins in the schizont stage of parasites [22]. In these two last studies, four glycolysis enzymes (enolase, aldolase, phosphoglycerate kinase and glyceraldehyde-3-phosphate dehydrogenase) and one heat shock protein (HSP 70 homolog) were commonly identified as up-regulated proteins under lumefantrine or chloroquine treatment, which was similar to the results seen under DOX treatment. The increased expression of redox metabolism proteins (1-Cys peroxiredoxin, 2-Cys peroxiredoxin) and 11 other "associated proteins" (Table 1 and Table 2), which have been recently shown to be potential targets of thioredoxin, glutaredoxin and plasmoredoxin [44], might represent another non-specific common feature of parasite responses to drug treatment.

**Figure 4:**
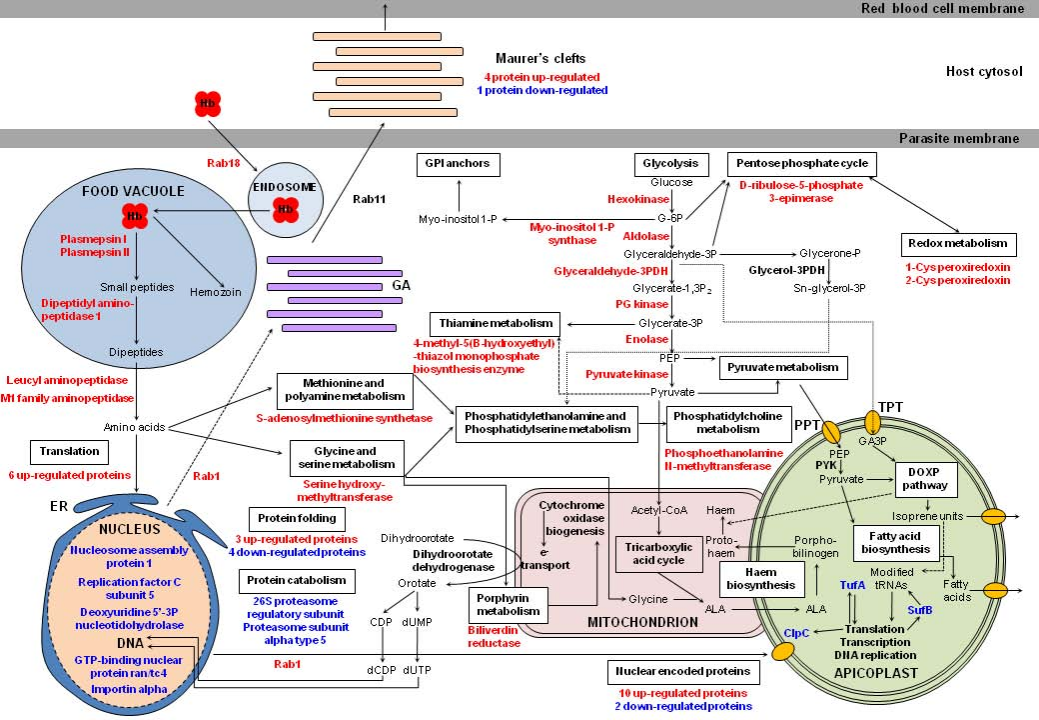
**Overview of metabolic pathways affected by doxycycline treatment in *P. falciparum *schizont stages adapted from Ginsburg, Hagai**. ''Malaria Parasite Metabolic Pathways'' http://sites.huji.ac.il/malaria/. Metabolic pathways are indicated by boxes and metabolic steps by arrows. Up- and down-regulated proteins under DOX treatment are indicated in red and blue, respectively. Abbreviations: ALA, aminolevulinic acid; DOXP, deoxyxylulose phosphate; ER, endoplasmic reticulum; GA, Golgi apparatus; GA3P, glyceraldehydes-3-phosphate; GPI, glycosyl phosphatidylinositol; Hb, haemoglobin; PEP, phosphoenolpyruvate; PDH, phosphate dehydrogenase; PG, phosphoglycerate; PPT, phosphoenolpyruvate transporter; PYK, pyruvate kinase; TPT, triose phosphate transporter.

Alternately, some metabolic pathways (Figure 4) might represent a specific response of parasites to DOX. Several studies have shown that tetracyclines, which are members of the DOX family, directly inhibit mitochondrial protein synthesis [13,45,46]. This inhibition would lead to a decrease in the mitochondrial respiratory chain activity [16] because the plasmodial mitochondrial genome encodes cytochrome *c *oxidase subunits I and III and apocytochrome *b*. The mitochondrial respiratory chain is coupled to dihydroorotate dehydrogenase activity, which has been shown to be depressed under tetracycline treatment [14]. This enzyme is involved in *de novo *pyrimidine biosynthesis, and its inhibition is associated with decreased levels of nucleotides and deoxynucleotides in *P. falciparum *in response to tetracycline treatment [15]. DOX inhibition of mitochondrial protein synthesis could be responsible for DNA replication impairment as suggested in the present study, *i.e*. replication factor C subunit 5 and deoxyuridine 5'-3P nucleotidohydrolase were down-regulated (Figure 4).

Two recent works have confirmed specific action by the cyclines on the *P. falciparum *apicoplast [17,18]; replication and transcription of the plastid genome as well as the import of nuclear encoded proteins into the plastid matrices' were inhibited in response to cycline treatment. Cyclines are assumed to inhibit plastid protein synthesis, but the precise mechanism of action has not yet been identified. The apicoplast genome encodes different tRNAs, rRNA, TufA (a translational elongation factor), SufB (involved in iron metabolism and modification of tRNAs [47]), ClpC (a protease required for nuclear-encoded protein import into the apicoplast) and a DNA dependent RNA polymerase (involved in transcription). This organelle is implicated in fatty acid and isoprenoid precursor synthesis and in heme biosynthesis in tight association with mitochondria [48]. In the present study, ClpC, TufA and SufB were found to be down-regulated under DOX treatment, but only at the transcriptional level. The inhibition of ClpC could explain the defect in protein import into the apicoplast and consequently the overexpression of 12 encoded nuclear proteins that are localized to the apicoplast (Figure 4).

## Conclusions

The present study has given the first insights into changes in protein regulation in *P. falciparum *upon DOX treatment, suggesting that *P. falciparum *apicoplasts and mitochondria are the targets of DOX. It has also confirmed that 2D-DIGE and iTRAQ are powerful and complementary techniques in studying protein changes in response to drug treatment. However, more experiments will be needed to characterize the specific molecular mechanisms of DOX treatment. In order to prove that DOX inhibits plastid or mitochondrial translational further biochemical approaches would probably be required.

## Abbreviations

DOX: doxycycline; RBCs: red blood cells; iRBCs: infected red blood cells.

## Competing interests

The authors declare that they have no competing interests.

## Authors' contributions

SB, LA, TF, CR and BP conceived and designed the experiments, SB, LA, MB, NW and AF performed the experiments, SB, LA, MB, EB, NW, AF and CR analyzed the data, MB, EB and SG contributed reagents/materials/analysis tools, and SB, LA, CR and BP wrote the paper.

## Supplementary Material

Additional file 1 **Methods**. Supplementary Methods Click here for file

Additional file 2 **SCX separation profiles of iTRAQ labelled peptides from the three biological replicates of soluble proteins at 214 nm**. Supplementary Figure Click here for file

Additional file 3 **Differentially expressed proteins in DOX-treated parasites identified from differential 2D-DIGE (pH 3-10, 4-7 and 6-11) analysis**. Supplementary Table Click here for file

Additional file 4 **Primer pairs used in quantitative RT-PCR**. Supplementary Table Click here for file

Additional file 5 **The 2D gel proteomic map of schizont stages of *P. falciparum***. Spots with a significant intensity change between doxycycline treatment and untreated are indicated by a circle in the soluble proteomic map with pI 3-10 (A), and in the membrane proteomic map with pI 4-7 (B) and pI 6-11 (C). (D) Enlargement of panel (A) to focus on differentially expressed protein spots following DOX treatment. Click here for file
