# Supplementary material for: Plasmodium falciparum proteome changes in response to doxycycline treatment
Source: Malar J. 2010 May 25;9:141. doi: 10.1186/1475-2875-9-141 (PMC2890676; doi:10.1186/1475-2875-9-141)
Supplement: Additional file 1 — Methods. Supplementary Methods [file 1475-2875-9-141-S1.DOC]

**Supplementary Methods**

**2D-DIGE**

Isoelectric focusing (IEF) was performed in 18-cm 3-10, 4-7 and 6-11 linear IPG strips (GE Healthcare). Destreak buffer (GE Healthcare) containing 1% (v/v) IPG buffer 3-10, 4-7 or 6-11 (GE Healthcare) was used for overnight rehydration of IPG strips in an immobiline dry strip reswelling tray (GE Healthcare). The samples were applied at the acidic end of the IPG strip using a cup-loading technique. IEF was carried out on an Ettan IPGphor II (GE Healthcare) electrophoresis unit at 20°C for a total of 48 kVh (ramp to 300 V in 3h, ramp to 1000 V in 6h, ramp to 8000 V in 3h, and hold at 8000 V for 4 h; 50 µA maximum per strip). Prior to separation in the second dimension, IPG strips were reduced and alkylated in a equilibration buffer containing 50 mM Tris-HCl, pH 8.6 buffer, 6 M urea, 2% SDS and 30% glycerol supplemented with 1% (w/v) DTT or 2.5% (w/v) iodoacetamide for 10 min. IPG strips were then placed on top of 12.5% uniform polyacrylamide gels poured between low fluorescence glass plates using an Ettan-DALT Six gel caster (GE Healthcare). Strips were overlaid with 0.5% agarose in 1X running buffer containing bromophenol blue, and the proteins were further separated by SDS-PAGE (15 W per gel) at 20°C in a Ettan DALT Six electrophoresis system (GE Healthcare) until the dye front migrated out of the gel.

**iTRAQ labelling and strong cation exchange**

iTRAQ-labelled peptides were loaded onto a PolySULFOETHYL A^TM^ column 100 mm × 2.1 mm containing 5-μm particles with a 200-Ǻ pore size (PolyLC Inc.) that was previously washed with buffer A (10 mM KH_2_PO_4_ and 25% v/v ACN, pH 3.0). The peptides were eluted at a flow rate of 200 µL/min with a gradient of 0-60% buffer B (10 mM KH_2_PO_4_, 500 mM KCl and 25% v/v ACN, pH 3.0) for 30 min followed by a holding step at 60% buffer B for 3 min and ramping up to 100% buffer B for 15 min.

**Protein identification by nano-LC MS/MS**

The gradient profile consisted of a linear gradient from 95% buffer A (H_2_O, 0.1% HCOOH) to 60% buffer B (80% ACN, 0.1% HCOOH) for 60 min followed by a linear gradient to 95% buffer B for 10 min. Mass data acquisitions were performed by MassLynx 4.0 software using automatic switching between MS and MS/MS modes. The internal parameters of Q-TOF were set as follows: the electro-spray capillary voltage was set to 3.2 kV; the cone voltage was set to 30 V; and the source temperature was set to 80°C. The MS survey scan was conducted at m/z 400-1300 with a scan time of 1 s and an interscan time of 0.1 s. When the intensity of a peak rose above a threshold of 15 counts, tandem mass spectra were acquired. Normalized collision energies for peptide fragmentation were set using the charge-state recognition files for +2 and +3 peptide ions. The scan range for MS/MS acquisition was from m/z 50 to 1500 with a scan time of 1 s and an interscan time of 0.1 s. Fragmentation was performed using argon as the collision gas and with the collision energy profile optimized for various mass ranges and charges of precursor ions. Mass data collected during a nano-LC-MS/MS analysis were processed using ProteinLynx Global Server 2.2 software (Waters) with the following parameters to generate peak lists in the micromass pkl format: no background subtraction, smooth 3/2 Savitzky Golay and no deisotoping. Pkl files were then entered into a local search engine Mascot Daemon v2.2.2 (Matrix Science). Purification and analysis of iTRAQ-labelled peptides were performed as described previously for protein identification, with the following minor modifications: after on line desalting of the sample for 20 min, the elution profile consisted of a linear gradient from 95% buffer A (H_2_O, 0.1% HCOOH) to 60% buffer B (80% ACN, 0.1% HCOOH) for 130 min followed by a linear gradient to 95% buffer B for 10 min. Normalized collision energies for peptide fragmentation were set using the charge-state recognition files for +2, +3 and +4 peptide ions with the collision energy profile increased by 20% compared to the optimized profile used without iTRAQ labelling. This modified profile gave a good, but not complete, fragmentation of the iTRAQ molecule and sufficient intensity of the reporter ions to perform quantification of the peptides.

**iTRAQ protein database search and quantification**

For Mascot distiller software (v2.1.1, Matrix Science), the default parameters were used except for the following: for MS processing, peak half width was fixed to 0.1; for MS/MS processing, precursor charge was used as a maximum; for the time domain, the minimum precursor mass was fixed at 400, the maximum precursor mass was fixed at 1,700 and the precursor m/z tolerance was 0.1; and for the peak picking, the maximum peak m/z was 10,000.

For protein identification, MS/MS data files were searched together to produce a merged output using the “merge MS/MS files into single search” option. Search parameters for peptide and MS/MS mass tolerance were both set at ± 0.8 Da; one missed cleavage made from the trypsin digest was allowed. Fixed modifications were iTRAQ (K), iTRAQ (N-terminal) and MMTS (C), and variable modifications were oxidation (M) and iTRAQ (Y). Peptides were considered identified if their MASCOT individual ion score was higher than the MASCOT identity scores (*p* < 0.01). A false discovery rate of 0% was obtained when the decoy database search was performed against a randomized decoy database created by MASCOT using identical search parameters and validation criteria.

For protein quantification, after the data conversion, an automated quantification of peptide abundance was performed by MultiQ software using selected, unique iTRAQ-labelled peptides with confident MS/MS identification (MASCOT score ≥ 30) and detected signature ions (*m/z* = 114, 115, 116, 117). For the detector dynamic range filter, signature peaks with ion counts higher than 1,000 counts or lower than 30 counts were filtered out by Multi-Q. To calculate average protein ratios, the ratios of quantified, unique iTRAQ non-degenerated peptides were weighted according to their peak intensities to minimize the standard deviation and automatically normalized by Multi-Q. Each quantified protein was manually validated, and only quantified proteins present in at least two of the biological triplicates were conserved for further analysis.
