# Supplementary figures and images for: Plasmodium falciparum proteome changes in response to doxycycline treatment
Source: Malar J. 2010 May 25;9:141. doi: 10.1186/1475-2875-9-141 (PMC2890676; doi:10.1186/1475-2875-9-141)

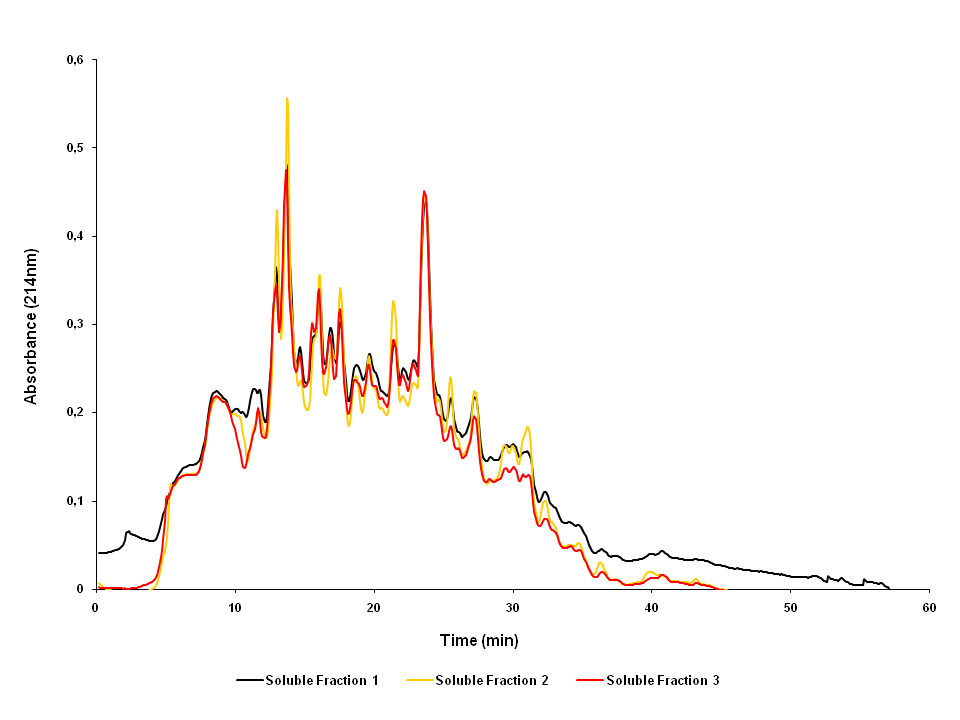

Supplement: Additional file 2 — SCX separation profiles of iTRAQ labelled peptides from the three biological replicates of soluble proteins at 214 nm. Supplementary Figure [file 1475-2875-9-141-S2.TIFF]

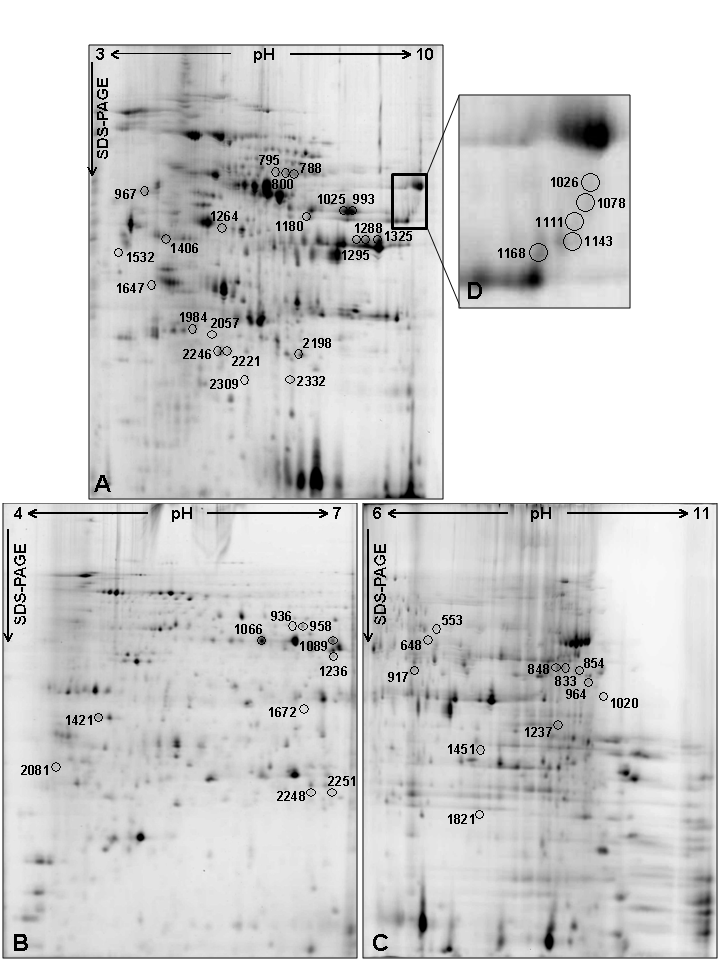

Supplement: Additional file 5 — The 2D gel proteomic map of schizont stages of P. falciparum. Spots with a significant intensity change between doxycycline treatment and untreated are indicated by a circle in the soluble proteomic map with pI 3-10 (A), and in the membrane proteomic map with pI 4-7 (B) and pI 6-11 (C). (D) Enlargement of panel (A) to focus on differentially expressed protein spots following DOX treatment. [file 1475-2875-9-141-S5.TIFF]
