# Supplementary material for: Plasmodium falciparum proteome changes in response to doxycycline treatment
Source: Malar J. 2010 May 25;9:141. doi: 10.1186/1475-2875-9-141 (PMC2890676; doi:10.1186/1475-2875-9-141)
Supplement: Additional file 3 — Differentially expressed proteins in DOX-treated parasites identified from differential 2D-DIGE (pH 3-10, 4-7 and 6-11) analysis. Supplementary Table [file 1475-2875-9-141-S3.RTF]

Table AF1. Differentially expressed proteins in DOX-treated parasites identified from differential 2D-DIGE (pH 3-10, 4-7 and 6-11) analysis.

PlasmoDB Accession Number	Name	Spot	Mass (Da)	pI	% Coverage	Peptides matched	Peptide Sequence	MASCOT score	
PF13_0304	Elongation factor 1 alpha	1078	49155.97	9.12	10	5	K.VVEENPK.A
K.FLNIDSK.I
K.QIVVGVNK.M
K.IGGIGTVPVGR.V
K.SGDSALVSLEPK.K	236	
PF13_0304	Elongation factor 1 alpha	1111	49155.97	9.12	8	4	K.VVEENPK.A
R.GYVASDTK.N
K.IGGIGTVPVGR.V
K.SGDSALVSLEPK.K	225.97	
PF13_0304	Elongation factor 1 alpha	1143	49155.97	9.12	10	5	K.VVEENPK.A
K.FLNIDSK.I
R.IPLQGVYK.I
K.IGGIGTVPVGR.V
K.SGDSALVSLEPK.K	245.64	
PF13_0304	Elongation factor 1 alpha	1026	49155.97	9.12	12	6	K.VVEENPK.A
K.FLNIDSK.I
R.IPLQGVYK.I
R.QTIAVGIIK.S
K.IGGIGTVPVGR.V
R.TLIEALDTMEPPK.R	308.89	
PF13_0304	Elongation factor 1 alpha	848	49155.97	9.12	23	9	K.VVEENPK.A
K.FLNIDSK.I
K.YAWVLDK.L
R.IPLQGVYK.I
K.IGGIGTVPVGR.V
R.TLIEALDTMEPPK.R
K.AGMVLNFAPSAVVSECK.S
K.EVLEEARPGDNIGFNVK.N
K.VDFIPISGFEGDNLIEK.S	411.19	
PF13_0304	Elongation factor 1 alpha	833	49155.97	9.12	11	6	K.VVEENPK.A
K.FLNIDSK.I
K.QIVVGVNK.M
R.IPLQGVYK.I
K.IGGIGTVPVGR.V
K.SGDSALVSLEPK.K	306.38	
PF13_0304	Elongation factor 1 alpha	1168	49155.97	9.12	12	6	K.VVEENPK.A
K.QIVVGVNK.M
R.IPLQGVYK.I
R.QTIAVGIIK.S
K.IGGIGTVPVGR.V
K.SGDSALVSLEPK.K	254.36	
PF13_0143	Phosphoribosylpyrophosphate synthetase	964	49864.72	9.39	7	3	R.VPISAADVAR.M
R.MIEAMGVDR.V
R.FADGEVSMQFLESIR.G	174.46	
PFB0445c	DEAD box helicase, UAP56	2246	52646.75	5.68	4	2	R.ILVSTDLFGR.G
R.FEVAISEMPNK.I	137.92	
PFI1105w	Phosphoglycerate kinase	1025	45569.16	7.63	24	10	K.FQNDLTK.L
R.AHSSMVGVK.L
K.ANKEDVEK.F
K.IVGEIMEK.A
K.ELPGVLALSNK.-
K.IGTSLFDEAGSK.I
R.MIIGGGMAYTFK.K
K.ENSVILLENLR.F
K.NVQIFLPVDFK.I
K.IADNFDNNANTK.F	514.51	
PFI1105w	Phosphoglycerate kinase	993	45569.16	7.63	13	6	K.FQNDLTK.L
R.AHSSMVGVK.L
K.IVGEIMEK.A
R.FHIEEEGK.G
K.IGTSLFDEAGSK.I
K.IADNFDNNANTK.F	342.62	
PF14_0598	Glyceraldehyde-3-phosphate dehydrogenase	1288	37068.08	7.59	6	2	K.ELASSHLK.G
R.VPIGTVSVVDLVCR.L	113.64	
PF14_0598	Glyceraldehyde-3-phosphate dehydrogenase	1295	37068.08	7.59	14	5	R.SSIFDMK.A
K.DPSQIPWGK.C
K.YEEVALEIK.K
K.AGLALNDNFFK.L
R.VPIGTVSVVDLVCR.L	276.02	
PF14_0598	Glyceraldehyde-3-phosphate dehydrogenase	1325	37068.08	7.59	25	8	K.LGINGFGR.I
K.VIMSAPPK.D
K.ELASSHLK.G
K.DPSQIPWGK.C
K.YEEVALEIK.K
K.AGLALNDNFFK.L
R.VPIGTVSVVDLVCR.L
K.QLIVSNASCTTNCLAPLAK.V	472.15	
PFI1090w	S-adenosylmethionine synthetase, putative	1236	45271.97	6.28	5	2	K.VACEVCAK.K K.FVLGGPAADAGLTGR.K	87.61	
PFF1300w	Putative pyruvate kinase	800	56480.36	7.50	8	4	K.LIDAGMDICR.F
K.EEVSGGTNLMK.V
R.GDLGMEISPEK.V
K.ILAESDGIMIAR.G	216.82	
PFF1300w	Putative pyruvate kinase	788	56480.36	7.50	6	3	K.EEVSGGTNLMK.V
R.GDLGMEISPEK.V
K.ILAESDGIMIAR.G	120.35	
PFF1300w	Putative pyruvate kinase	1672	56480.36	7.50	4	2	K.VGSFQGTDIVIR.N
K.ILAESDGIMIAR.G	109.98	
PFF1300w	Putative pyruvate kinase	553	56480.36	7.50	6	3	K.NMNLPNVK.V
K.VGSFQGTDIVIR.N
R.QILEPNNVNLR.S	149.29	
PF14_0368	2-Cys peroxiredoxin	1821	21964.40	6.65	12	2	R.NVELIGCSVDSK.Y
R.SYNVLFGDSVSLR.A	116.26	
PFE0690c	Rab1 protein	2221	24129.11	6.89	17	4	R.TITSAYYR.G
R.DYDYLYK.I
K.IILIGDSGVGK.S
K.LQIWDTAGQER.F	171.35	
PF11_0396	Protein phosphatase 2C	2309	108211.09	4.40	2	2	K.ESLDGGNLELDPSR.Y
K.LDEEMLLSENQEK.L	144.42	
PFF1335c	4-methyl-5(B-hydroxyethyl)-thiazol monophosphate biosynthesis enzyme	2198	20564.67	6.95	28	5	K.IVEHLLGR.Q
K.SEQVCLQSK.N
K.NVVLADTTISK.V
R.AGVHVTTASVEK.S
R.NNIYDVLVIPGGMK.G	282.17	
MAL13P1.283	TCP-1/cpn60 chaperonin family, putative	958	58505.82	6.09	13	6	K.IDDTVICR.-
K.TDMDNTVVVK.D
K.GGVIITNDGATILK.E
R.ASNNLMLDEAER.S
R.DAVNDLALDFLAK.A
K.AVLPGGAAPEMELSQK.L	314.57	
MAL13P1.283	TCP-1/cpn60 chaperonin family, putative	936	58505.82	6.09	28	13	K.DYNSMDR.L
K.IDDTVICR.-
K.TDMDNTVVVK.D
K.DTNVDLNNVR.I
K.ISESFFEASLK.S
K.LATETVMMILK.I
R.EMSIPIDLNDK.N
K.GGVIITNDGATILK.E
R.ASNNLMLDEAER.S
K.IGLIQFCLSLPK.T
R.DAVNDLALDFLAK.A
K.IASTGCNLLIIQK.S
K.AVLPGGAAPEMELSQK.L	828.27	
PFF1155w	Hexokinase	795	56081.03	6.72	5	3	R.ATNDPVEGR.D
K.LMNDAFVR.A
R.DQETYSLK.F	123.11	
PF14_0425	Fructose-bisphosphate aldolase	1180	40479.03	8.33	28	10	K.VLSCVFK.A
R.TVLVIDTAK.G
K.LENTIENR.A
R.AEANSLATYGK.Y
R.YASICQQNR.L
K.STQGLDGLAER.C
K.TTTQDVGFLTVR.T
K.GILAADESTQTIK.K
K.GLVNIPCTDEEK.S
K.GGAGGENAGASLYEK.K	552.05	
PF14_0425	Fructose-bisphosphate aldolase	917	40479.03	8.33	28	10	K.VLSCVFK.A
R.TVLVIDTAK.G
K.LENTIENR.A
R.AEANSLATYGK.Y
R.YASICQQNR.L
K.STQGLDGLAER.C
K.TTTQDVGFLTVR.T
K.GILAADESTQTIK.K
K.GLVNIPCTDEEK.S
R.ALQASVLNTWQGK.K	561.16	
PF14_0076	Plasmepsin 1 precursor	1406	51656.32	6.72	10	4	K.GYLTIGGIEDR.F
R.FYEGQLTYEK.L
K.DIVTIANLSFPYK.F
K.DLSIGSVDPVVVELK.N	215.45	
MAL8P1.69	14-3-3 protein	1647	29856.49	4.96	7	2	K.LAEQAER.Y
K.LIPNTSESESK.V	71.13	
PF08_0131	1-Cys peroxiredoxin	2251	25054.52	6.38	11	2	K.ATVLYPATTGR.N
K.CCILPSVDNADLPK.L	83.46	
PF08_0074	DNA/RNA-binding protein Alba, putative	1237	27298.84	10.58	12	3	R.MTNYVNYGAK.I
K.DAGYQPPLDEK.Y
K.TVSFIEILLSR.E	112.95	
PF14_0486	Elongation factor 2	1264	85034.03	6.30	5	4	R.GIVISEEQK.L
R.GAGQIMPACK.K
K.EGVLCEENMR.G
K.AYPLAEGLPEAIDK.N	163.92	
PF13_0214	Elongation factor 1-gamma	1984	50998.52	8.84	3	2	R.LENNFSK.Y
K.LDINNTQDK.K	77.32	
PF10_0155	Enolase	1066	48989.39	6.21	17	8	K.YNQLLR.I
K.YLAQLAGK.K
K.TYDLDFK.T
K.NINEIIAPK.L
K.NACNALLLK.V
K.LIGMNCTEQK.K
K.IDNLMVEELDGSK.N
R.IEESLGNNAVFAGEK.F	438.55	
PF10_0155	Enolase	1089	48989.39	6.21	24	10	K.YNQLLR.I
K.YLAQLAGK.K
K.TYDLDFK.T
K.NINEIIAPK.L
K.NACNALLLK.V
K.LIGMNCTEQK.K
K.IDNLMVEELDGSK.N
R.IEESLGNNAVFAGEK.F
K.IAMDVAASEFYNSENK.T
R.AAVPSGASTGIYEALELR.D	583.9	
PFL0960w	D-ribulose-5-phosphate 3-epimerase, putative	2248	25646.19	6.07	9	2	K.LAEETQR.M
K.AIIAPSVLASNISK.L	93.86	
PF11_0117	Replication factor C subunit 5	1020	41005.26	9.04	6	2	R.TLETYIR.N
K.DAEFLSEGAQAGLR.R	106	
PF08_0109	Proteasome subunit alpha type 5, putative	2057	55546.45	4.73	4	2	K.LDGTCDIIR.S
R.FIECPEYENNLSK.F	78.67	
PFL0185c	Nucleosome assembly protein 1, putative	967	42198.67	4.90	9	4	K.TVQQTVNR.D
R.EALVGNGEAK.I
K.EYYDYESK.F
K.MTDLTEEQK.E	169.5	
PF11_0313	60S ribosomal protein P0	2081	34069.99	6.56	9	2	K.GVSNVAALSR.A
K.MVENPEAFAAVAAPASAAK.A	99.64	
PF11_0282	Deoxyuridine 5'-triphosphate nucleotidohydrolase	2332	19675.06	6.53	31	5	K.DEVLKPK.S
K.IVCLSDEVR.E
R.GEGGFGSTSNNK.Y
R.GEGGFGSTSNNKY.-
R.LANSIGLIDAGYR.G	306.15	
MAL8P1.95	Conserved Plasmodium protein	1532	37933.22	4.13	10	3	K.IDDEGQFEK.D
K.FDTEGQIVSLK.-
K.NLENNEDDETNVGR.N	240.9	
PF14_0678	Exp-2	1421	33159.74	5.30	11	3	R.QDPSLIVAK.I
R.EIVGDNTIEK.K
K.YEFDVDSSETDSTK.D	152.53	
PF11_0183	GTP-binding nuclear protein ran/tc4	1451	24973.81	7.72	11	2	K.LILVGDGGVGK.T
R.VCETIPMVLVGNK.V	97.88	
PF13_0033	26S proteasome regulatory subunit	854	45047.76	9.05	24	8	R.FSQGTSADR.E
K.LGDIDYESVCR.L
R.VSLDMTTLTVMK.R
R.NVCTEAGMFAIR.A
K.ALQSVGQIIGQVLK.Q
R.AMASNINCNFMR.I
K.VNYNQIGGLSEQIR.Q
R.EVVELPILNPYLYK.R	341.1	
PF14_0359	HSP40, Subfamily A	648	49179.66	6.81	9	4	K.NCTTDEVK.K
R.GEDIVSEVK.V
R.EVLDEGMPTYK.D
K.ILVNCTNSGFIR.H	203.96	
