# Supplementary material for: Plasmodium falciparum proteome changes in response to doxycycline treatment
Source: Malar J. 2010 May 25;9:141. doi: 10.1186/1475-2875-9-141 (PMC2890676; doi:10.1186/1475-2875-9-141)
Supplement: Additional file 4 — Primer pairs used in quantitative RT-PCR. Supplementary Table [file 1475-2875-9-141-S4.RTF]

Table AF2. Primer pairs used in quantitative RT-PCR.
Accession nr	Forward	Reverse	
PFCOMPIRB-TufA	AGGTACTATAGGGCATGTAGATCA	CTGGAGCTGAATCAATATCTG	
PFCOMPIRB-SufB	TCGATTATAACATGGAAATACCCTTCT	ACCAGTATCAGCTATTTGCATATTTGA	
PFCOMPIRB-ClpC	GGTACTGGTAAAACTGAATTAGCAAAAA	AACCAACATAACCAGGAGGTGAAC	
PFI1090w	CTGGAAAATTTGTTCTTGGAGGA	TGAGCTCCCCATCCACCATA	
MAL8P1.95	CAGTTATTCATTTATCAAGGGCCTG	TTGTAGCATCCATTTCCGTCTTC	
PF14_0439	ATTACATTCGATTCAGGAGGATACAA	CGGCTGCACATCCACTCATAT	
PF07_0033	GTGAGGAATGACATATCCGAAAGG	GGTGAAACCAACTAATGTCGGG	
PFE1195w	CGATAGTCAGGAGTTATATGACATAGGTG	CCAACACGATCTAAGGACTCTTCAC	
